# Digital health to strengthen pediatric surgical referral and follow-up in Low-resource settings: progress, gaps, and future directions

**DOI:** 10.3389/fsurg.2026.1835282

**Published:** 2026-06-09

**Authors:** Xiaoyi Chen, Xuchen Luo, Jiehua Deng

**Affiliations:** 1 Department of Plastic and Aesthetic Surgery, The Second Affiliated Hospital of Guilin Medical University, Guilin, China; 2 School of Clinical Medicine, Xiangnan University, Chenzhou, China; 3 School for Economics, Zhejiang University of Science & Technology, Hangzhou, China

**Keywords:** digital health, follow-up, low-resource settings, pediatric surgery, referral

## Abstract

Timely referral and effective postoperative follow-up remain major challenges in pediatric surgical care in low-resource settings, where geographic barriers, workforce shortages, fragmented communication, and weak health-system coordination often disrupt continuity of care. Digital health has emerged as a potentially practical approach to addressing these pathway failures through tools such as teleconsultation, mobile messaging, image sharing, and remote postoperative contact. This mini review examines current applications of digital health in pediatric surgical referral and follow-up, with particular attention to their emerging value, implementation barriers, and future directions. Existing studies suggest that relatively simple digital tools may improve referral coordination, facilitate specialist input, reduce unnecessary travel, support caregiver communication, and strengthen postoperative continuity. However, the available evidence remains uneven and is still dominated by pilot studies, small observational designs, and context-specific implementation experiences. Important concerns also persist regarding infrastructure constraints, digital inequity, workflow integration, privacy, governance, and long-term sustainability. Current research has focused more heavily on postoperative follow-up than on referral and triage, leaving important parts of the pediatric surgical pathway underexplored. Overall, digital health shows genuine promise for strengthening pediatric surgical systems in low-resource settings, but its impact will depend on equitable implementation, stronger evaluation, and integration into routine care pathways rather than isolated technological adoption.

## 1 Introduction

Timely access to pediatric surgical care remains a major challenge in many low-resource settings. For children with congenital anomalies, injuries, abdominal emergencies, or other surgically treatable conditions, delayed referral and weak postoperative follow-up can lead to avoidable complications, prolonged disability, and poorer long-term outcomes (1, 2). Recent reviews of pediatric surgery in low- and middle-income countries have consistently emphasized that the main barriers to better outcomes extend beyond operative capacity itself (3, 4). They also include weak referral systems, limited continuity of care, shortages of trained personnel, and fragile health-system coordination (5).

Referral and follow-up are especially important in pediatric surgery because they shape the full pathway of care rather than a single clinical encounter (6, 7). Many children first present to peripheral facilities where specialist surgical expertise is limited, diagnostic uncertainty is common, and transfer decisions may be delayed (8–10). After surgery, families frequently face long travel distances, transport costs, income loss, and limited access to structured postoperative review, all of which increase the risk of missed follow-up (11). In low-resource settings, these problems are magnified by long waiting times, fragmented communication between facilities, and limited systems for tracking patients across levels of care.

Against this background, digital health has gained increasing attention as a practical means of strengthening communication, coordination, and continuity across the surgical care pathway (12, 13). The World Health Organization frames digital health not as a stand-alone technological solution, but as an approach that should be integrated into broader health-system functions and service delivery (14–16). In this review, digital health is used as the broader umbrella term referring to the use of digital technologies to support health services, communication, information exchange, and care delivery. Telemedicine is treated as a narrower component within digital health and refers more specifically to the remote delivery of clinical support, consultation, follow-up, or decision-making through communication technologies. In the context of pediatric surgical referral and follow-up, most of the literature reviewed here concerns telemedicine-enabled activities such as remote consultation, image sharing, messaging, telephone contact, and postoperative review. To avoid conceptual ambiguity, we therefore use digital health as the overarching term and refer to telemedicine when discussing specific remote clinical interactions.

The rapid expansion of digital health and telemedicine was also accelerated by the COVID-19 pandemic, which pushed many health systems to adopt remote consultation, communication, and follow-up models more quickly than had previously been the case (17, 18). Although much of this acceleration was documented in higher-resource settings, the pandemic also highlighted the practical value of remote care pathways when in-person access was disrupted. In this sense, COVID-19 did not create the need for digital health in pediatric surgical care, but it made the importance of flexible, remote, and continuity-oriented care models more visible.

In surgical care across low- and middle-income countries, telemedicine is already emerging in areas such as consultation, coordination, and especially postoperative follow-up (19). Scoping reviews further suggest that relatively simple tools, including telephone calls, two-way texting, file sharing, and video communication, are often the most feasible modalities in resource-constrained settings.

In pediatric surgical care, such tools may offer several practical advantages. They can help frontline providers obtain specialist input more quickly, support triage and referral decisions, reduce unnecessary travel, and maintain postoperative contact with families who might otherwise be lost to follow-up. Telemedicine-based wound review and remote postoperative contact have also been discussed as useful adjuncts for identifying early complications and improving continuity after discharge, particularly where in-person return visits are difficult to complete. However, enthusiasm for these approaches should be balanced by recognition of their limits. Existing evidence remains uneven, many interventions are pilot-based, and important concerns persist regarding infrastructure, digital exclusion, workflow integration, privacy, and sustainability.

This mini review examines how digital health is being used to strengthen pediatric surgical referral and follow-up in low-resource settings. It focuses on the current forms of digital application and their emerging value, while also assessing persistent implementation challenges, unresolved evidence gaps, and priority directions for future research and practice. By treating digital health as part of a broader effort to improve access, continuity, and system responsiveness, this review aims to clarify where digital approaches are genuinely promising and where expectations remain ahead of evidence.

## 2 Methods

This mini review used a focused narrative search to identify literature on digital health and telemedicine in pediatric surgical referral and postoperative follow-up, with particular attention to low-resource settings. We searched PubMed, Scopus, and Google Scholar for English-language publications using combinations of terms including “digital health”, “telemedicine”, “telehealth”, “mobile health”, “pediatric surgery”, “referral”, “triage”, “follow-up”, “postoperative care”, and “low-resource settings” or “low- and middle-income countries”. The search was primarily focused on publications from January 2015 to February 2026, while a small number of earlier or broader background references were retained when they were important for conceptual framing or health-system context.

We included review articles, observational studies, pilot studies, feasibility studies, and relevant policy or systems papers if they addressed digital communication, remote consultation, referral coordination, or postoperative follow-up in pediatric surgery or closely related pediatric specialty-care contexts. We excluded studies that were clearly unrelated to pediatric populations, unrelated to surgical pathways, or focused on digital health applications without relevance to referral or follow-up. Given the limited pediatric surgery-specific literature, evidence synthesis was narrative rather than quantitative, and studies were grouped thematically around referral support, postoperative follow-up, caregiver communication, implementation barriers, and system-level implications.

A total of 41 publications were included in the final narrative synthesis. The included literature spanned high-income settings, low- and middle-income settings, and mixed or multi-setting contexts. Because a substantial proportion of the evidence came from systematic reviews, broader specialty-care literature, and multi-country or cross-context discussions, the distribution of country settings was described narratively rather than reduced to a rigid numerical breakdown by income category.

Table 1 summarizes the main characteristics of selected key studies included in this mini review, including study design, setting, population of interest, digital modality, and main relevance to referral or follow-up. The included evidence spans high-income, low- and middle-income, and mixed or multi-setting contexts.

**Table 1:** Summary of key studies included in this mini review.

Study	Study design	Setting and income context	Population of interest	Digital modality	Main relevance to referral or follow-up
O'Dwyer et al., 2024 (27)	Original hospital-based evaluation	Specialised acute care hospital, high-income setting	Children requiring specialty care, referring providers	eConsult and referral technology	Shows how digital referral tools can improve access to specialist pediatric care and strengthen coordination across levels of care
Hartasanchez et al., 2022 (25)	Systematic review	Mixed settings	Patients, caregivers, clinicians	Telemedicine-supported shared decision-making	Supports the role of remote consultation in clarifying decisions and communication during referral and care planning
Bamgboje-Ayodele et al., 2024 (24)	Service evaluation	Urgent care collaboration context	Frontline providers and tertiary services	Advice lines and remote consultation	Illustrates how telecommunication pathways can improve early triage and inter-service collaboration
Yanti et al., 2025 (26)	Narrative/multidisciplinary review	Multiple settings	Health systems and providers	Electronic referral systems	Highlights gains in coordination, efficiency, and traceability in referral pathways
Nguyen et al., 2022 (30)	Systematic review	Mixed settings, including LMIC-relevant literature	Pediatric surgical patients and caregivers	Mobile health, phone follow-up, text messaging, image sharing, video contact	Core pediatric surgery evidence showing that digital postoperative follow-up is feasible and increasingly used after discharge
Baniasadi et al., 2023 (31)	Pilot study	Post-discharge surgical care setting	Postoperative surgical patients	Mobile application for wound telemonitoring	Demonstrates feasibility of app-based wound monitoring after discharge and supports the broader logic of remote complication surveillance
Rochon et al., 2025 (22)	Review	Mixed surgical settings	Surgical patients and care teams	Smartphone-based wound monitoring	Supports the use of image sharing and remote wound review for early postoperative surveillance
Vivas-Colmenares et al., 2025 (41)	Systematic review	Mixed settings	Parents and caregivers of pediatric patients	Telemedicine for postoperative caregiver education	Shows that remote education may improve caregiver knowledge, confidence, and preparedness after discharge
Gudipudi et al., 2024 (43)	Systematic review	Mixed pediatric surgical subspecialty settings	Parents, providers, and pediatric patients	Telehealth follow-up and outpatient teleconsultation	Reports generally favourable satisfaction with telehealth in pediatric surgical care, while noting dependence on encounter quality and technology reliability
Abdelmohsen, 2026 (42)	Cross-sectional study	Single-center pediatric surgery follow-up setting	Parents/caregivers	Telemedicine follow-up	Provides direct evidence that caregivers often report high satisfaction with postoperative telemedicine follow-up
Campbell et al., 2023 (40)	Systematic review	Mixed settings	Patients and families	Virtual consultations	Supports the broader conclusion that virtual care can reduce travel burden, waiting time, and logistical costs
Bella and Stathopoulos, 2026 (39)	Health services/transport study	Mixed caregiver-facing healthcare settings	Caregivers	Virtual vs. in-person care pathways	Reinforces the argument that remote follow-up may reduce indirect household burden such as travel and time costs

## 3 Digital health applications and emerging value in pediatric surgical referral and follow-up

Existing research on digital health in pediatric surgical care remains limited, but several recurring themes can already be identified. Current studies are concentrated less on highly advanced digital platforms and more on practical tools that can be deployed within constrained systems, especially teleconsultation, mobile phone communication, image sharing, video-based review, and digitally supported postoperative follow-up (20, 21). Across the broader surgical literature in low- and middle-income countries, telemedicine has been used most often for postoperative review, consultation, and patient education, with basic modalities such as telephone calls and two-way messaging appearing more feasible than resource-intensive digital infrastructure (22, 23). This pattern is important for pediatric surgery, where the main challenge is often not the absence of technological innovation itself, but the need for workable solutions that can support communication and continuity under conditions of workforce shortage and geographic fragmentation.

A first line of research focuses on digital support for referral and triage. In many low-resource settings, children with surgical conditions initially present to district hospitals or peripheral clinics where pediatric surgical expertise is unavailable. Studies and reviews in telemedicine and referral systems suggest that telemedicine-enabled communication between frontline providers and specialists can improve decision-making at this early stage by enabling remote assessment, clarifying urgency, and reducing uncertainty around transfer (24, 25). In the broader specialty-care literature, mobile health screening linked to telemedicine referral has been shown to improve access to specialty services in underserved settings, while electronic referral research has emphasized gains in coordination, efficiency, and traceability across levels of care (26, 27). Although pediatric surgery-specific evidence remains relatively sparse, the same logic applies: telemedicine-enabled tools may improve referral quality by shortening the interval between first presentation and specialist input, especially where the conventional pathway is slow, fragmented, or dependent on informal communication (28, 29).

A second and more developed line of research concerns digital postoperative follow-up, which appears to be the most common application in the available surgical literature. In these studies, the population of interest is not always the same. Some papers focus primarily on children as postoperative patients, particularly in relation to complication review, wound assessment, and the need for in-person reassessment, whereas others focus more directly on caregivers when evaluating communication, satisfaction, understanding, and the practical feasibility of remote follow-up after discharge. A scoping review of telemedicine in surgical care in low- and middle-income countries found that postoperative visits were the most frequent use case, often supported by simple methods such as telephone calls, text messaging, file sharing, and video communication (30, 31). Pediatric surgical studies from higher-resource settings also provide useful proof of concept. Randomized and observational studies have reported that telemedicine-based postoperative follow-up can reduce time burden, save travel, and maintain satisfactory clinical review for selected patients, while cohort data suggest that many concerns can be managed remotely, with only a subset of children requiring in-person reassessment (32, 33). This literature is not directly generalizable to all low-resource settings, but it provides an important starting point for thinking about how remote follow-up might function where return visits are costly or difficult to complete.

A third strand of literature focuses explicitly on caregivers rather than children alone and highlights caregiver communication and education as an essential component of digital follow-up. In pediatric surgery, postoperative recovery depends heavily on what caregivers understand and do after discharge, including wound care, symptom recognition, medication adherence, feeding practices, and timely help-seeking (34, 35). Recent reviews on telemedicine for parent and caregiver support after pediatric surgery suggest that remote communication may strengthen caregiver knowledge, confidence, and satisfaction, even though the evidence base is still emerging and heterogeneous. This point is particularly relevant in low-resource settings, where discharge counseling may be brief, return travel may be difficult, and families may have limited access to immediate professional advice once the child has left the hospital (36–38). Under these conditions, digital follow-up is not only a matter of convenience; it may also function as a practical extension of postoperative education and safety monitoring.

A fourth theme in the literature concerns the perceived value of digital health for access, efficiency, and family burden. Studies in pediatric surgery and related surgical settings have repeatedly reported that virtual contact can reduce travel, lower indirect costs, shorten waiting times, and improve convenience for families (39, 40). This is especially important for pediatric patients because attendance commonly requires the presence of a caregiver and may involve missed work, lost wages, school disruption, and wider household costs. At the level of reported outcomes, child-centered endpoints are more often clinical, whereas caregiver-centered outcomes are more often related to knowledge, confidence, satisfaction, convenience, and burden. Research on caregiver perceptions in pediatric surgery has also found generally favorable views of telemedicine, although satisfaction is shaped by the quality of the encounter and by the reliability of the technology used (41–43). Taken together, these studies suggest that digital health may create value not only by preserving contact with clinicians, but also by reducing the broader economic and logistical burden associated with referral and follow-up.

Despite these encouraging findings, the present literature remains uneven in scope and depth. Much of the evidence comes from small pilots, single-center studies, or settings outside the lowest-resource environments. Pediatric surgery-specific evidence remains substantially thinner than the broader telemedicine literature, and referral pathways have received less attention than postoperative follow-up (30, 41, 43). Moreover, many existing studies prioritize feasibility, satisfaction, or uptake rather than stronger endpoints such as reduced referral delay, improved follow-up completion, cost-effectiveness, complication detection, or long-term outcomes (44). The current evidence therefore points to genuine promise, but it does not yet support overly broad claims about effectiveness across all contexts. At this stage, the strongest conclusion is that digital health appears most useful when applied to specific pathway problems, particularly specialist access, caregiver communication, and continuity after discharge, rather than when framed as a universal solution to pediatric surgical system weakness.

## 4 Challenges, evidence gaps, and future directions

Despite growing interest in digital health for pediatric surgical referral and follow-up, the existing literature also reveals several persistent challenges that limit implementation and weaken the strength of current conclusions. One of the most frequently noted barriers is the fragility of the underlying service environment. Reviews of telemedicine in low- and middle-income countries repeatedly show that digital interventions are constrained by unstable internet access, poor network coverage, unreliable electricity, limited device availability, and the absence of interoperable information systems. These barriers are especially consequential in pediatric surgery because referral and follow-up depend on timely communication across facilities and with caregivers, meaning that even simple digital pathways can fail when the supporting infrastructure is inconsistent. Broader WHO guidance similarly emphasizes that digital health cannot perform effectively when basic system prerequisites remain weak.

A second major concern is digital inequity. Much of the available literature presents digital health as a means of improving access, yet several studies also caution that the same interventions may reproduce or even deepen exclusion if they assume reliable phone ownership, stable connectivity, high digital literacy, or consistent caregiver availability. In low-resource settings, these conditions cannot be taken for granted. Families with lower income, low literacy, rural residence, or unstable access to communication technology may be the very groups most likely to benefit from better referral and follow-up systems, yet also the most likely to be left out of digitally mediated models. Reviews in global digital health have therefore stressed that questions of equity should be treated as central design considerations rather than secondary concerns. For pediatric surgery, this issue is particularly important because children depend on caregivers to receive, interpret, and act on digital communication.

A third challenge concerns workflow integration and workforce readiness. Existing studies suggest that digital tools are most promising when they are embedded in routine clinical pathways, but many reported interventions remain pilot-based, externally supported, or insufficiently integrated into everyday practice. Frontline staff may need additional training to use teleconsultation platforms, manage image-based review, document remote interactions, and coordinate follow-up across facilities. Where staffing is already constrained, digital systems may be perceived not as efficiency-enhancing tools but as additional administrative burdens. The broader implementation literature repeatedly shows that technological adoption in low-resource settings depends not only on usability, but also on governance, workflow alignment, leadership support, and the fit between the intervention and local service organization. This helps explain why feasibility can appear promising in pilot studies while sustained routine use remains difficult.

Data governance and privacy present a further unresolved issue. In practice, low-cost communication tools such as messaging applications, image sharing, and informal mobile communication may be attractive because they are readily available and familiar to both providers and families. However, studies and policy guidance have raised concerns about confidentiality, secure storage, consent, and accountability when patient information is transmitted outside formal health information systems. These questions are especially sensitive in pediatric care because clinical information concerns minors and often involves parental or caregiver mediation. Yet many studies of digital follow-up emphasize acceptability and convenience without providing equally detailed discussion of data protection standards, governance arrangements, or legal responsibilities. This creates an important tension within the literature: the most feasible tools may also be the least formally governed.

The current evidence base also remains limited in important methodological respects. Much of the literature on digital health in surgical care, and especially in pediatric contexts, is based on small observational studies, feasibility reports, single-center pilots, or narrowly defined implementation experiences. Outcomes commonly reported include satisfaction, acceptability, and feasibility, whereas stronger endpoints such as referral timeliness, completion of follow-up, avoidable transfer reduction, complication detection, cost-effectiveness, and longer-term clinical outcomes are assessed much less consistently. Reviews of telemedicine in surgery have similarly concluded that the overall evidence base remains heterogeneous and often insufficient for strong causal claims. As a result, the present literature supports cautious optimism, but not broad generalizations about effectiveness across settings, patient groups, or health-system contexts.

Another important gap is the imbalance between postoperative follow-up and referral research. Existing studies more commonly examine digital contact after surgery than digital support for early triage and referral decision-making. This is understandable, since follow-up is easier to operationalize and often more feasible to study. However, from a health-systems perspective, delayed or poorly coordinated referral may be just as consequential as incomplete follow-up, especially in settings where specialist pediatric surgical expertise is geographically concentrated. The literature therefore remains skewed toward one part of the care pathway, leaving the upstream stages of recognition, referral initiation, transfer coordination, and specialist access comparatively underexplored. This imbalance suggests that future research should move beyond discharge-centered models and examine the entire pathway from first presentation to postoperative recovery.

These limitations point toward several priorities for future work. First, there is a clear need for stronger implementation research that examines how digital tools can be integrated into existing referral pathways, workforce routines, and health information systems rather than assessed only as stand-alone pilots. Second, future studies should use more comparable and policy-relevant outcome measures, including referral delay, successful specialist linkage, follow-up completion, family cost burden, complication identification, and sustainability over time. Third, equity-sensitive evaluation should become a standard feature of study design. Research should identify not only who benefits from digital referral and follow-up, but also who is excluded, under what conditions, and why. Finally, greater attention should be given to scalability and governance. The key question is no longer whether digital contact is technically possible, but whether it can be delivered safely, equitably, and sustainably at system level in the settings where pediatric surgical access is most fragile. These priorities are consistent with WHO calls for digital health to be evidence-based, integrated, and system-oriented rather than purely technology-driven.

Taken together, the current literature suggests that digital health has genuine potential to strengthen pediatric surgical referral and follow-up, but that promise remains conditional. The main challenge is not simply expanding digital contact. It is designing models that can function within constrained infrastructures, fit routine clinical workflows, protect vulnerable patients, and reduce rather than reproduce inequity. Future progress will therefore depend less on technological novelty than on implementation quality, governance, and alignment with broader efforts to strengthen pediatric surgical systems.

## 5 Discussion

The current literature suggests that digital health has real but conditional value in pediatric surgical referral and follow-up. Its greatest strength lies in addressing pathway failures that are common in low-resource settings, particularly delayed specialist input, weak communication between facilities, and loss to follow-up after discharge. Across the broader surgical literature in low- and middle-income countries, telemedicine is already being used most often for postoperative care, consultation, and patient education, with telephone calls and two-way texting emerging as especially feasible approaches. This pattern is important because it shows that the most relevant innovations in these settings are often not the most technologically advanced, but the ones most compatible with constrained infrastructures and everyday clinical workflows. At the same time, WHO guidance has consistently framed digital health as a means of strengthening health systems rather than a stand-alone technical fix, which is a useful lens through which to interpret the pediatric surgical evidence. Importantly, the literature does not always study the same unit of analysis. Some studies evaluate children as patients, some evaluate caregivers as the main users of follow-up communication, and some examine provider-facing referral systems. This distinction matters because the expected benefits and the most appropriate outcome measures differ across these groups.

From this perspective, the central contribution of digital health is not that it replaces conventional pediatric surgical care, but that it can improve connectivity across weak parts of the care pathway. In many low-resource environments, poor outcomes are shaped not only by shortages of surgeons, anesthetists, or infrastructure, but also by failures of coordination between first presentation, referral, specialist review, discharge, and postoperative monitoring. Digital tools may therefore be most valuable when they are used to reduce fragmentation across these stages. This interpretation is also consistent with recent work in global pediatric surgery, which increasingly treats access, quality, and outcomes as system-level problems rather than challenges confined to operative technique alone.

At the same time, the existing evidence does not justify overly broad claims about effectiveness. Much of the literature remains dominated by feasibility studies, small pilots, and observational reports, while stronger evidence on comparative effectiveness, cost-effectiveness, longer-term outcomes, and scale-up remains limited. Even where remote follow-up appears practical, it is still unclear which patients can be safely managed at a distance, which outcomes are most appropriate for evaluation, and how digital models should be integrated into referral governance and routine documentation. The literature therefore supports cautious optimism rather than technological enthusiasm. Digital health appears promising, but promise alone is not enough to establish policy readiness.

A further implication concerns equity. Digital models are often promoted as mechanisms for extending access, yet they may also exclude the very populations most affected by weak pediatric surgical systems if implementation assumes reliable device access, stable connectivity, high digital literacy, or uninterrupted caregiver availability. This tension is not peripheral; it is central to the future of digital referral and follow-up in low-resource settings. Equitable integration requires attention not only to technical usability, but also to affordability, caregiver burden, language, literacy, and the possibility that informal digital communication may create new forms of inconsistency or vulnerability. More broadly, recent work on equitable digital integration in global health has argued that digital tools should be designed around the realities of underserved populations rather than layered onto systems in ways that privilege those already easiest to reach.

These observations point toward a more realistic agenda for future work. The most important next step is likely not the creation of increasingly sophisticated technologies, but the development of implementation models that are simple, context-sensitive, and compatible with routine care. Future research should move beyond narrow demonstrations of feasibility and instead evaluate whether digital referral and follow-up improve meaningful pathway outcomes such as reduced delay, better specialist linkage, fewer unnecessary transfers, improved follow-up completion, earlier complication detection, and lower family burden. Standardized outcome frameworks would also make evidence more comparable across studies. Finally, digital health should be studied as part of pediatric surgical system strengthening, not as an isolated intervention. That means linking research on technology with questions of workforce, governance, financing, data systems, and long-term service organization.

Overall, the literature indicates that digital health can make pediatric surgical referral and follow-up more responsive, more continuous, and potentially more equitable in low-resource settings. Yet its success depends far less on technological novelty than on whether it is embedded within functioning clinical pathways, supported by appropriate governance, and evaluated with outcomes that matter to children, caregivers, and health systems. In that sense, digital health should be understood not as a substitute for pediatric surgical capacity, but as one component of a broader effort to make that capacity more reachable, coordinated, and sustainable.

## 6 Conclusion

Digital health is emerging as a practical means of strengthening pediatric surgical referral and follow-up in low-resource settings. Existing studies suggest that relatively simple tools, particularly telephone-based communication, messaging, teleconsultation, and remote postoperative contact, may help improve referral coordination, sustain follow-up, reduce family burden, and strengthen continuity of care. These functions are especially valuable in settings where pediatric surgical pathways are weakened by geographic distance, workforce shortages, fragmented communication, and fragile service organization.

At present, however, the evidence remains uneven and should be interpreted with caution. Much of the literature is still based on small-scale or context-specific studies, with stronger evidence lacking for long-term outcomes, cost-effectiveness, equity effects, and system-level sustainability. Digital health should therefore not be viewed as a substitute for pediatric surgical workforce, infrastructure, or referral governance. Its greatest potential lies in supporting these systems by making communication more timely, care pathways more connected, and follow-up more feasible for children and caregivers.

Future progress will depend on whether digital interventions can move beyond isolated pilots and become safely, equitably, and sustainably integrated into routine pediatric surgical care. In this sense, the key challenge is no longer whether digital tools can be used, but how they can be embedded into broader efforts to improve access, continuity, and outcomes across pediatric surgical systems.
